# Photocatalytic Technology for Palm Oil Mill Effluent (POME) Wastewater Treatment: Current Progress and Future Perspective

**DOI:** 10.3390/ma14112846

**Published:** 2021-05-26

**Authors:** Wibawa Hendra Saputera, Aryan Fathoni Amri, Rahman Daiyan, Dwiwahju Sasongko

**Affiliations:** 1 Research Group on Energy and Chemical Engineering Processing System, Department of Chemical Engineering, Faculty of Industrial Technology, Institut Teknologi Bandung, Jl. Ganesha No. 10, Bandung 40132, Indonesia; aryanfathoni@gmail.com (A.F.A.); sasongko@che.itb.ac.id (D.S.); 2 Center for Catalysis and Reaction Engineering, Institut Teknologi Bandung, Jl. Ganesha No. 10, Bandung 40132, Indonesia; 3 Research Center for New and Renewable Energy (PPEBT), Institut Teknologi Bandung, Jl. Ganesha No. 10, Bandung 40132, Indonesia; 4 Particles and Catalysis Research Group, School of Chemical Engineering, Faculty of Engineering, The University of New South Wales, Sydney, NSW 2052, Australia; r.daiyan@unsw.edu.au

**Keywords:** palm oil mill effluent, treatment technologies, photocatalytic degradation, photocatalyst, wastewater treatment

## Abstract

The palm oil industry produces liquid waste called POME (palm oil mill effluent). POME is stated as one of the wastes that are difficult to handle because of its large production and ineffective treatment. It will disturb the ecosystem with a high organic matter content if the waste is disposed directly into the environment. The authorities have established policies and regulations in the POME waste quality standard before being discharged into the environment. However, at this time, there are still many factories in Indonesia that have not been able to meet the standard of POME waste disposal with the existing treatment technology. Currently, the POME treatment system is still using a conventional system known as an open pond system. Although this process can reduce pollutants’ concentration, it will produce much sludge, requiring a large pond area and a long processing time. To overcome the inability of the conventional system to process POME is believed to be a challenge. Extensive effort is being invested in developing alternative technologies for the POME waste treatment to reduce POME waste safely. Several technologies have been studied, such as anaerobic processes, membrane technology, advanced oxidation processes (AOPs), membrane technology, adsorption, steam reforming, and coagulation. Among other things, an AOP, namely photocatalytic technology, has the potential to treat POME waste. This paper provides information on the feasibility of photocatalytic technology for treating POME waste. Although there are some challenges in this technology’s large-scale application, this paper proposes several strategies and directions to overcome these challenges.

## 1. Introduction

Waste is a material produced from industrial or domestic (household) activities whose existence is often undesirable because it negatively impacts the environment. There are three types of waste, including solid waste, liquid waste, and gas waste, which can be classified into inorganic and organic waste. Based on the economic value, waste can be classified into waste that has economic value and does not have economic value. Waste that has economic value is waste that can be further processed to produce added-value products. Economic growth causes an increase in industrial activities and is a magnet for population movements increasing industrial and domestic waste. The increase of waste produced is proportional to an increase in a country’s gross domestic product (GDP). The sectors contributing the most to Indonesia’s GDP include manufacturing, agriculture, forestry and fishery, wholesale and retail trade, construction, mining, and quarrying. Based on the Indonesian statistical centers, forestry is the second largest contributor to GDP after manufacturing [1]. The forestry sector produces waste from three stage processes, including preharvest, harvest, and post-harvest processes. In Indonesia, one of the promising business sectors in the forestry fields is the oil palm industry.

**Figure 1:**
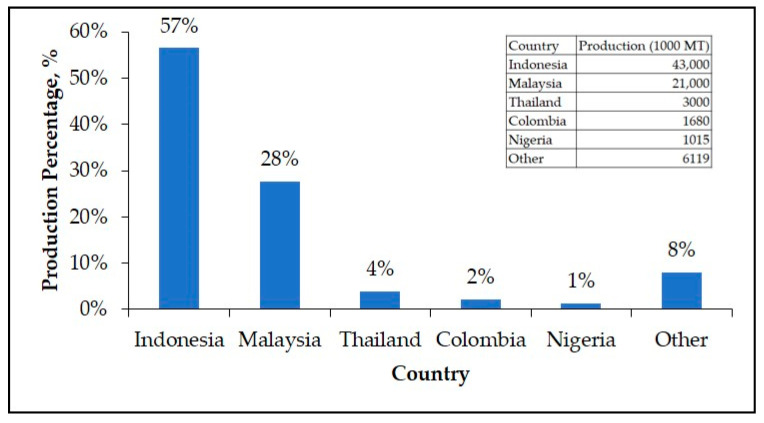
Comparison of palm oil production in 2019. Adapted from ref. [7].

**Figure 2:**
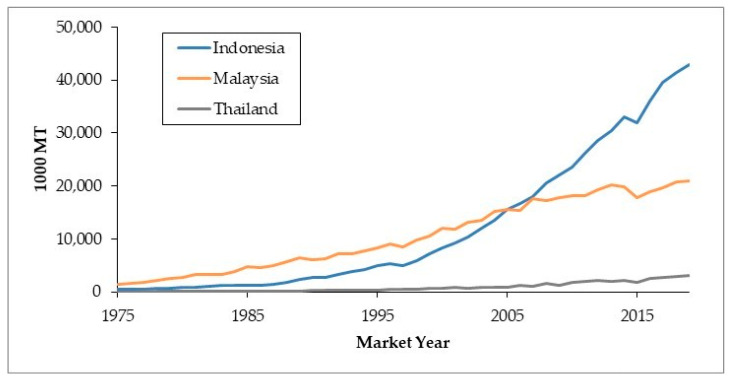
Comparison of palm oil production in Indonesia, Malaysia, and Thailand from 1975 to 2018. Adapted from ref. [7].

As the world’s largest crude palm oil (CPO) producer shown in Figure 1, Indonesia has the world’s largest palm oil land. Based on the Indonesian plantation statistics in 2018, the total area of palm oil in Indonesia reached 14.67 million hectares with palm oil production of 42.87 million tons [2]. Based on Figure 2, it is clearly shown that there is an enhancement trend of the palm oil production in Indonesia, Malaysia, and Thailand, which commonly utilize as cooking oil and biodiesel.

The palm oil industry produces liquid waste called POME (palm oil mill effluent). Each ton of CPO production produces approximately 2.5–3.0 m^3^ of POME [3]. POME is stated as one of the wastes that are difficult to handle because of its large production and ineffective existing treatment. It is a waste with the lowest amount of fiber content among all the wastes of palm oil processes [4]. POME contains a high organic load that causes high biochemical oxygen demand (BOD, 10,000–44,000 mg/L) and chemical oxygen demand (COD, 16,000–100,000 mg/L) [5]. With a high organic matter content, the pollutants’ levels will be higher to negatively affect the ecosystem if the waste is discharged directly into the environment. During the processing of POME, the odor will emerge. Besides that, POME also has a brownish appearance, where the surrounding environment will be interrupted by POME disposal. A higher production level has also increased the volume of untreated POME that has been discharged from the processing mills [6]. The authorities have established policies and regulations in the POME waste quality standard before being discharged into the environment.

Nevertheless, many factories in Indonesia have not met the standard of POME waste disposal with the existing treatment technology. Currently, the POME treatment system is still using a conventional system known as an open pond system. The system includes anaerobic ponds, aerobic ponds, and settling ponds. The principle of this system is to use microorganisms to degrade organic pollutant compounds in POME. This system operates simply but will produce much sludge, requiring a large pond area and long processing time.

**Figure 3:**
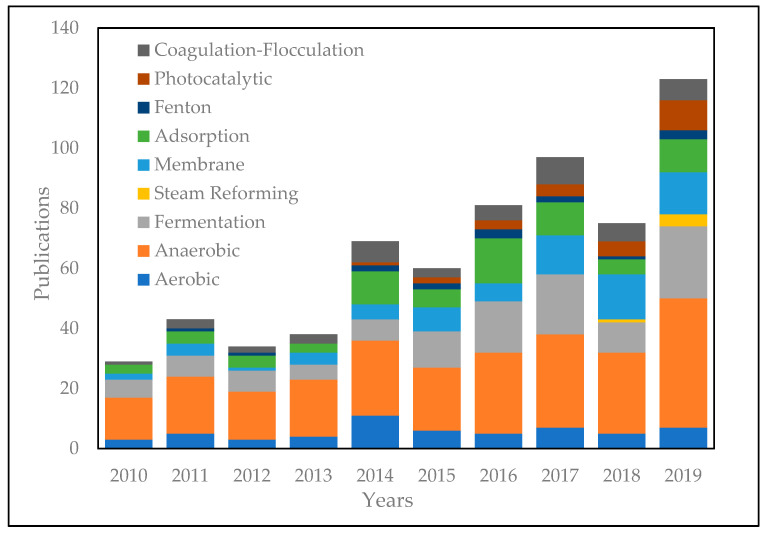
The number of annual publications related to POME treatment technologies in the past ten years. Indexed by Scopus (TITLE-ABS-KEY (terms); terms: aerobic, anaerobic, fermentation, steam reforming, membrane, adsorption, Fenton, photocatalytic, ozonation, and coagulation-flocculation for palm oil mill effluent).

To overcome the inability of the conventional system to process POME is believed to be a challenge. Many studies have been conducted to find other alternative technologies for POME waste treatment to reduce POME waste to a safe level of BOD 100 mg/L [8]. Several technologies have been reported, namely biological treatment, physiochemical treatment, thermochemical treatment, and integrated treatment [9]. The graph outlining data on the number of publications from 2010 to 2019 related to POME treatment using various technologies is presented in Figure 3. Although these alternative technologies have shown satisfying results in high-quality output waste, all of these processes are not yet feasible to replace open pond systems because they require high processing costs. As it is known that palm oil processing requires low costs to be competitive internationally, new technologies with high operating costs will not be attractive to palm oil mills [10].

For instance, physiochemical treatment, namely ultrafiltration membrane separation, can reduce pollutant elements by up to 90% with water [11]. However, this technology is not feasible for treating POME waste because the turbidity characteristics of POME cause membrane fouling. It is necessary to add a specific chemical that will inevitably increase processing costs to overcome fouling. For biological treatments, the general principle is similar to an open pond system, in which biological treatments have a long processing time [12,13]. Physiochemical treatments such as adsorption also still need further study [14], the absence of scalability studies [15], and also requires high maintenance costs due to the use of adsorbents on treating POME [10]. POME processing with thermochemical treatment such as catalytic steam reforming can also be used to process POME and also produced byproducts in the form of useful syngas [16,17,18]. As explained above, POME is composed of organic elements; thus, promising side products can be generated. However, the production of H_2_ syngas is still lower than conventional hydrocarbon reforming [16]. In addition, POME containing a high-water content (>95%) causes high energy requirements [10].

Another alternative technology is the advanced oxidation process (AOP) using heterogeneous photocatalytic in semiconductors such as TiO_2_, ZnO, WO_3_, SnO_2_, CdS, SiO_2_, ZrO_2_, ZnO, Nb_2_O_3_, Fe_2_O_3_, V_2_O_5_, Sb_2_O_4_, CeO_2_, etc. [10]. In general, the general principle of AOP is producing hydroxyl radicals acting as a strong oxidizer that will react with organic compounds (pollutants) in waste converted into H_2_O and CO_2_ and other compounds, which are more biodegradable and harmless products [19]. Photon energy (UV light/visible light) is used as a driving force to activate semiconductor-based materials that act as a catalyst to degrade pollutants. This technology is environmentally friendly and classified as cost-effective in processing various pollutants such as organic and inorganic wastes [20]. More importantly, this technology can utilize natural sunlight as photon energy, reducing operational costs [21]. This technology is feasible to replace conventional open pool systems.

This paper will discuss some of the developments in existing POME processing technology, particularly photocatalytic technology. This review consists of four main parts: general information and the standard quality of POME waste, the development of technologies in POME processing including conventional and alternative technologies, the general principle of photocatalysis process and the development of photocatalysts including semiconductor-based and modification of semiconductor-based that are used for POME degradation, and operational parameters that affect the process and kinetic models of photocatalytic degradation of POME.

## 2. Characteristic of POME

Almost all methods in processing oil palm require the use of excess water. [5] Thani et al. reported that to process 1 ton of fresh fruit bunches (FFB), up to 1.5 m^3^ of water is needed, and as much as 50% of it ends up as POME waste. [3] Hasanudin et al. also reported that each production of 1 ton of CPO produced 2.5–3.0 m^3^ of POME. In previous studies conducted by [22], it was reported that 0.5–0.75 tons of POME produced in each processing of 1 ton of FFB.

The three main sources of POME waste come from the sterilizer condensate stage (17%), decanter or sludge separator stage (75%), and hydrocyclone waste stage (8%) [23]. POME is a colloidal suspension produced from a mixture of condensate sterilization, sludge separator, and hydrocyclone wastewater in a ratio of 9:15:1 [24]. POME has a high BOD and COD, which can cause pollution in the environment. COD is the amount of oxygen needed to oxidize organic substances present in wastes. The higher the level, it indicates that these substances are still in abnormal quantities and are dangerous if directly circulated to the surrounding environment. BOD is the amount of oxygen needed by bacteria to break down the organic waste. The higher the level, it indicates that the bacteria require much oxygen to reduce the waste. High COD and BOD levels can cause the death of the water population due to reduced oxygen levels.

POME is a brownish thick viscous liquid with a temperature between 80 and 100 °C at its output caused by the sterilization process and is acidic with a pH between 3.4 and 5.2. During palm oil production, there is no addition of chemicals; therefore, POME waste is a non-toxic waste [25]. However, POME can pollute the environment because it can reduce the content of dissolved oxygen in the water. POME waste disposal has various characteristics depending on processing techniques and raw materials’ quality, including age and fruit type [26]. Table 1 presents a summary of the differences in the characteristics of POME in Indonesia and Malaysia.

## 3. Laws and Legislations for POME Discharge

With the rapid development of palm oil production and increasing public awareness of environmental pollution, the palm oil industry is socially and legally obliged to treat its waste before being discharged into the environment.

In 1991, to prevent this waste’s negative effects, the Government of Indonesia made regulations regarding POME disposal standards into the environment. Since the regulation was passed, the palm oil industry must process their POME waste before releasing it into the environment. The standard limits on the quality of POME waste in Indonesia are summarized in Table 2. The latest regulation in 2014 stated that the COD standard is limited to 350 mg/L at the initial stage at 500 mg/L. Likewise, in the initial stages, the BOD standard is limited to 250 mg/L and then updated in 2014 to a lower concentration of 100 mg/L.

## 4. Conventional Palm Oil Mill Effluent (POME) Treatment Technologies

The content of POME waste consists of biodegradable organic matter. It is required to establish a POME processing system at each palm oil mill and strictly control the POME standard limits before discharged into the surrounding environment to avoid POME waste’s negative effects. It was reported by Rahayu et al. [30] that almost all palm oil mills in Indonesia use an open pond system in treating POME waste since the system has advantages from an economic point of view and the ease of operation.

**Figure 4:**
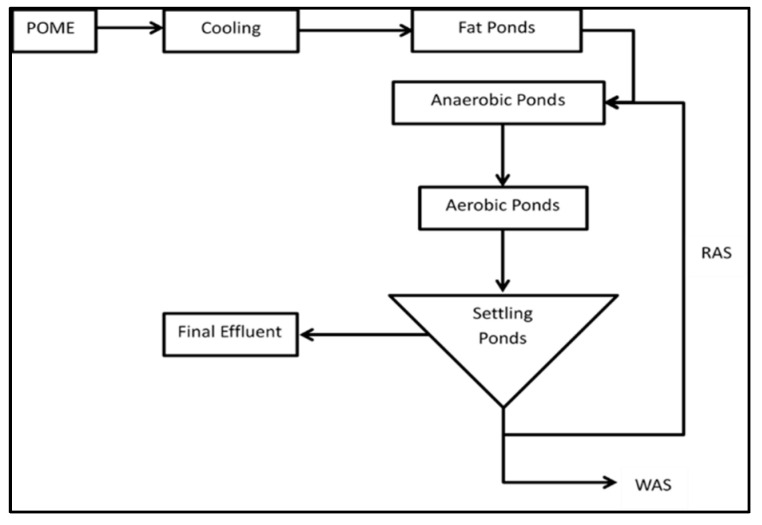
POME treatment scheme using conventional technology (open pond system). Adapted from ref. [30].

There are series of ponds with several treatments in the POME waste treatment process. This system consists of five types of ponds: cooling ponds, fat ponds, anaerobic ponds, aerobic ponds, and settling ponds (Figure 4). Each plant may give different naming and pool functions. The cooling pond serves to reduce the POME temperature ranging from 80 to 90 °C to reach the optimal temperature in the process of the next pool. As shown in Table 1, POME waste contains oil and grease, the remaining oil and fat in POME will be collected in a fat pool. The fat pool consists of a baffle pit or sump that can hold wastewater for 10 h. The most effective process requires a hydraulic retention time (HRT) of around 1–2 days [5].

Furthermore, POME will undergo processing in anaerobic and aerobic ponds. Organic substances in POME can be effectively degraded both in anaerobic and aerobic processes. Anaerobic can occur without oxygen, while aerobics takes place when there is oxygen content. According to Perez et al. [31], the most suitable method for POME processing is the anaerobic process. Usually, the anaerobic pool’s depth ranges from 5 to 7 m to minimize and prevent oxygen entry through the photosynthesis process. According to Yacob et al. [32], an anaerobic pool has a typical size (length × width × depth) with a processing capacity of 7500 m^3^ POME with a total HRT of 40 days is 60.0 m × 29.6 m × 5.8 m, respectively. However, the pond’s size depends on the palm oil mill’s production capacity and the land available for processing ponds [33]. After the anaerobic pool, POME is further processed in the aerobic pool. Aerobic pools usually have a depth of 1–1.5 m. It is intended that the transfer of oxygen (O_2_) can be evenly distributed throughout the pond [10]. The addition of oxygen is carried out by stirring or diffusion of air. Before being discharged into the environment, the aerobic pond’s waste is first deposited in a settling pond. In the sedimentation ponds, the mud will be descended and accumulated at the bottom of the pond.

Although the open pond system is economical, this system requires a longer retention time (20–60 days) and a more extensive area [24,34,35]. The open pond system also produces large amounts of mud that must be disposed of and processed further. This system also cannot completely decolorize POME waste [36]. Besides, there is methane gas production in the anaerobic process, released freely into the air. Chan et al. and Fujihara et al. [13,37] stated that methane release could contribute up to 70% of the total greenhouse gas emissions in the entire production process at the palm oil mill. Recently, technology in biogas can capture methane gas, reduce greenhouse gas emissions, produce renewable energy, and improve soil quality. However, the system is still too expensive to be commercialized [38]. Therefore, to overcome these issues, several alternative technologies have been developed, which will be discussed in the following section.

## 5. Alternative Palm Oil Mill Effluent (POME) Treatment Technologies

Many researchers have developed other alternative technologies to treat POME waste to overcome the open pool system’s weakness. These alternative technologies are developed to treat POME waste to reach quality standards and environmentally safety and expected to produce renewable energy. The last few years alternative technology has been widely studied like membrane technology [39], adsorption [40,41], coagulation-flocculation [42,43], AOP [44,45], and various anaerobic and aerobic degradation [46,47]. Although the research is still on a laboratory scale, the technology has shown more satisfying results than conventional systems or open ponds. The advantages and disadvantages of each technology will be explained in detail in the following section.

### 5.1. Biological Treatment

An open pond system is an economical and simple treatment method that can reduce the high pollution burden on POME [48]. Perez et al. [31] explained that the most suitable method for processing POME is a biological treatment, which is the anaerobic process. However, biological treatment using an open pool system has many weaknesses and can also cause other sources of pollutants such as methane (CH_4_) and hydrogen sulfide (H_2_S).

Many researchers have developed this system to overcome its weaknesses, such as shortening HRT, minimizing the land used for processing, and taking advantage of new sources of pollutants generated in the anaerobic process. For instance, the use of pollutants such as methane gas (CH_4_) can be further utilized to generate electricity [49]. Additionally, sludge production in open pond systems can be used as a fertilizer source [50].

**Figure 5:**
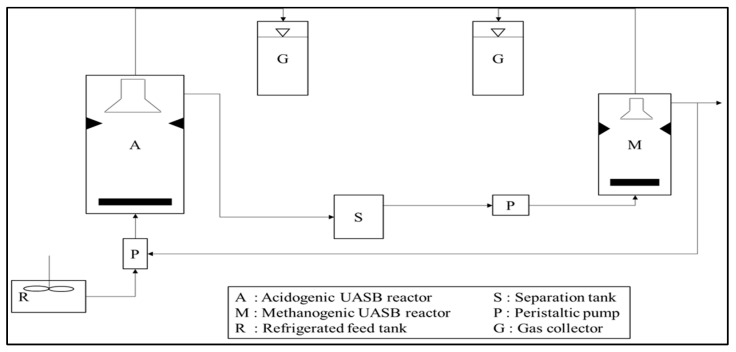
Schematic diagram of the two-stage UASB system. Adapted with permission from ref. [51]. Copyright 1996 Elsevier.

**Figure 6:**
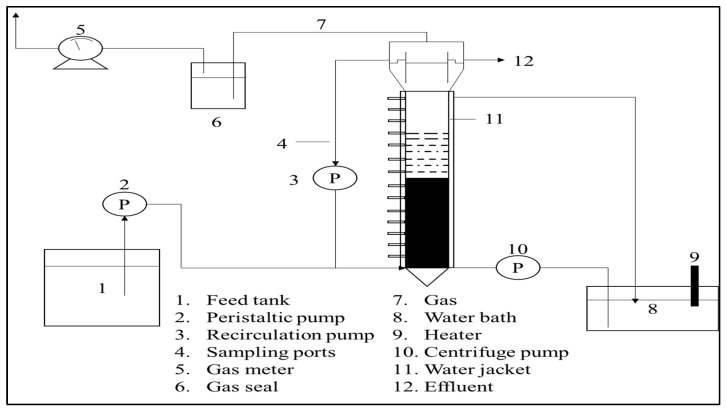
Schematic diagram of anaerobic EGSB reactor treating POME. Adapted with permission from ref. [52]. Copyright 2008 Elsevier.

**Figure 7:**
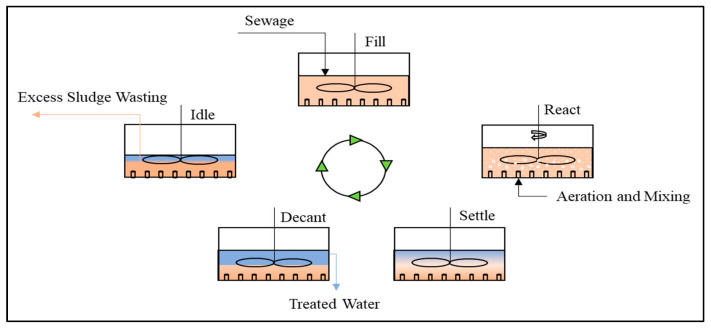
Process steps of SBR in one basin. Adapted with permission from ref. [53]. Copyright 2010 Elsevier.

**Figure 8:**
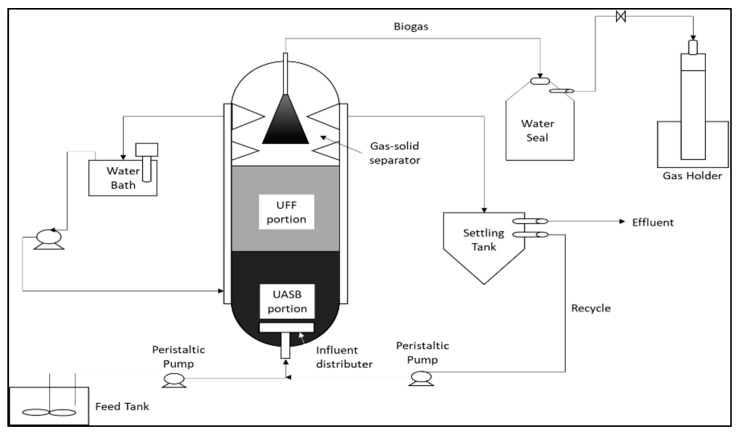
Schematic diagram of the UASFF reactor. Adapted with permission from ref. [54]. Copyright 2006 Elsevier.

In order to improve process performance and reduce HRT, several developments in biological treatments have been studied, such as up-flow anaerobic sludge blanket (UASB) (Figure 5) [51], expanded granular sludge bed (EGSB) (Figure 6) [52], sequencing batch reactors (SBR) (Figure 7) [53], up-flow anaerobic sludge fixed film reactor (UASFF) (Figure 8) [54], and rotating biological contactor (RBC) [55]. Although this technology can improve process efficiency and reduce HRT, most of these technologies have not yet been implemented on a large scale.

POME also contains high levels of organic acids, carbohydrates, lipids, minerals, and proteins, which can function as growth nutrients for microorganisms which are suitable substrates for hydrogen production using two stage sequential dark and photo fermentation. The yield of hydrogen from the first stage operation (dark-fermentation) was 0.78 mL H_2_/mL POME, then increased to 2.86 mL H_2_/mL POME after photo-fermentation under light and COD removal also increased from dark-fermentation (57%) until after photo-fermentation (93%). However, high light intensity decreases the photosynthetic activity of bacteria, causing a decrease in the yield of hydrogen. The low hydrogen yield is due to reduced nitrogenase activity, which is the only enzyme responsible for photo-fermentation.

### 5.2. Thermochemical Treatment

Thermochemical treatment is one technique for converting wet biomass into useful products such as syngas. Steam reforming is one example of this technology [17,18]. Syngas with hydrogen gas-rich was successfully produced from steam reforming of POME waste using a Ni-based catalyst [18]. The use of a catalyst can increase COD and BOD removal. It can also be increased by increasing liquid-hourly-space-velocity (LHSV). However, the increase in LHSV will cause carbon deposition on the catalyst’s surface, affecting lower H_2_ gas production. Energy consumption and clean energy income in steam reforming technology of POME waste to syngas must still be further analyzed to ensure such treatment’s feasibility.

**Figure 9:**
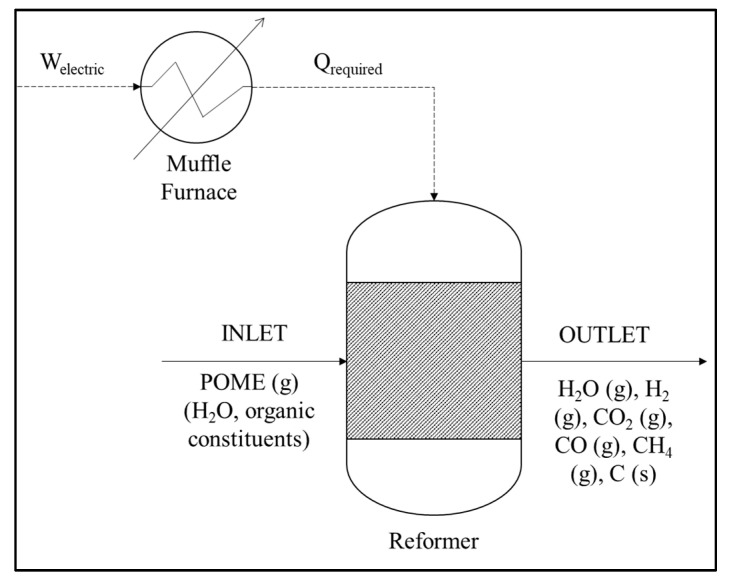
Process flow diagram of POME steam reforming. Adapted with permission from ref. [16]. Copyright 2019 Elsevier.

In work by Cheng et al. [56], the syngas production rate of LaNiO_3_-catalyzed steam reforming from POME is optimized concerning the POME flow rate, catalyst weight, and particle size. With a net acidity, synthesized LaNiO_3_ catalyzes POME vapor formation by breaking down large compounds and making simpler intermediates into syngas. At a higher POME flow rate (0.05–0.09 mL/min), greater POME partial pressures encourage the steam formation and water–gas shift, which increases catalytic performance. Beyond the optimal flow rate (0.09 mL/min), the coke-forming Boudouard reaction worsens catalytic activity. Catalytic performance was boosted for a longer residence time at a higher catalyst weight (0.1–0.3 g); nonetheless, the agglomerated catalyst was reduced when catalyst weight > 0.3 g. Finer LaNiO_3_ (particle size > 74 μm) with greater surface area to volume ratio exhibited better performance; however, ultrafine LaNiO_3_ (particle size < 74 μm) had poor performance because of occluded pores. Figure 9 illustrates the POME steam reforming process’s flow diagram, showing that the entire reactor setup is basically a reformer equipped with a muffle furnace.

**Figure 10:**
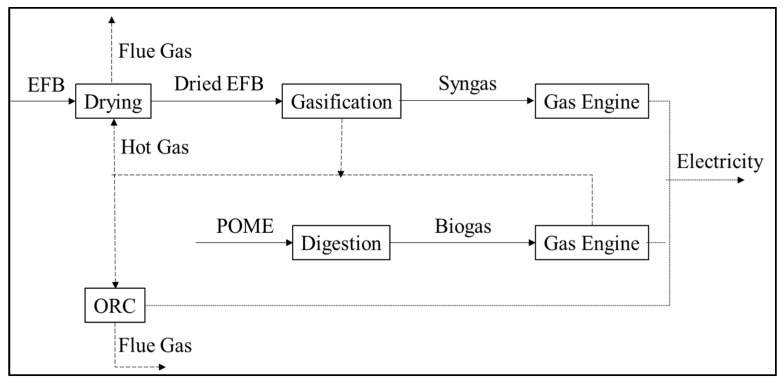
Conceptual diagram of a small-scale integrated power plant for EFB and POME. Adapted with permission from ref. [57]. Copyright 2017 Elsevier.

Besides, POME can be converted into biogas as fuel for electricity generation. The utilization of solid (EFB) and liquid (POME) from the palm oil milling process for power plants is proposed by Aziz et al. [57]. The proposed system consists of EFB gasification, POME digestion, and additional ORC (organic Rankine cycle) modules (Figure 10). The cogeneration system, which produces electricity and heat, produces syngas and biogas from both modules. Additionally, excess and unused heat from the system is converted into electricity through additional ORC modules. The total power generated and the power plant’s efficiency were 8.3 MW and 30.4%, respectively.

### 5.3. Physiochemical Treatment

Some physiochemical treatment has been developed for the POME waste treatment. The physiochemical treatment has a broad scope, consisting of physical treatment and chemical treatment, including coagulation–flocculation [58], membrane technology [59], adsorption [15], and integrated technology [60].

The coagulation–flocculation process is generally used as a pretreatment method used in POME waste treatment to remove suspended solids and residual oil [58]. Inorganic coagulants such as aluminum sulfate (alum) are widely used in waste treatment. Although this coagulant has proven to be effective, it is expensive and can produce dangerous sludge due to increased metal concentrations. As an alternative, natural coagulants are derived from animals or plants, such as seed gum. Research conducted by Shak et al. [58] combined the use of alum with seed gum. The results showed decreased total suspended solids and COD removal, respectively 81.58% and 48.22%. However, the efficiency of the treatment is not much higher than using alum alone. In the coagulation-flocculation process, it is only effective in reducing suspended solids, whereas COD is not significantly reduced. In addition, the resulting sludge is a concern because sludge treatment requires high costs. Apart from the low COD removal, the coagulation–flocculation technique cannot be used appropriately for POME processing due to operating and maintenance costs. On a lab scale, the coagulation–flocculation process was performed using jar test (VELP Scientifica Flocculator JLT4) in 500-mL beakers filled with 300 mL of palm oil mill effluent for each test run.

**Figure 11:**
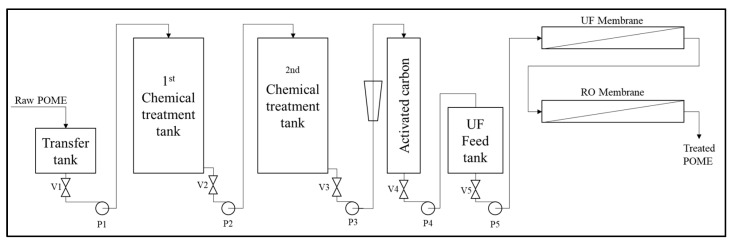
Schematic diagram of membrane technology for POME treatment. Adapted with permission from ref. [59]. Copyright 2003 Elsevier.

Membrane technology has been widely used in water and wastewater treatment and has been applied in various industry types. A POME treatment system based on membrane technology shows a high potential for eliminating the environmental problem, and also, this alternative treatment system offers water recycling. Membrane separation technology for treating POME has never been applied on an industrial scale due to POME characteristics, e.g., membrane processes have some limitations in dealing with the high suspended solids effluent. The membranes will suffer from fouling and degradation during use. Research conducted by Ahmad et al. [59] has two main treatment stages: pre-treatment and membrane separation. The pretreatment process consists of two stages of chemical treatment (coagulation, flocculation, and sedimentation) and active carbon treatment; for the treatment of membrane separation, UF and RO membranes are used to refine treated water further. A simplified flow diagram of the process is shown in Figure 11. The pretreatment process is necessary to remove the high content of suspended solids and oil in POME that would otherwise severely foul the membrane and lead to a shorter membrane life. The pretreatment process removed organic matter and suspended solids in POME by 97.9%, with a turbidity of 56% in COD and 71% in BOD. The promising results from the pretreatment process will reduce the membrane fouling phenomenon and degradation in flux. The turbidity value was reduced to almost 100% for the membrane separation treatment, with reduction in COD and BOD to be 98.8% and 99.4% respectively. In addition to fouling caused by this technology (membrane separation treatment), the use of this pretreatment will increase operating costs.

Adsorption has also been widely used to remove suspended solids [61], heavy metal [62], and residual oil [63] from POME waste. Many materials have been studied as potential adsorbents for POME treatment, such as chitosan, activated carbon (AC), natural zeolite, and bentonite. Ahmad et al. [61] conducted research to remove residual oil in POME around 6000 mg/L using three different adsorbents, namely chitosan, bentonite, and activated carbon. The jar-test method has been used to identify the best adsorbent for removing residual oil from POME. Chitosan shows the best removal among other adsorbents. These adsorbents can successfully remove 99% of residual oil and minimize the content of suspended solids respectively up to 25 mg/L (chitosan), 35 mg/L (activated carbon), and 70 mg/L (bentonite) under optimum conditions. As with coagulation and flocculation, this technique is only as effective at removing suspended solids, heavy metals, and oil residues.

Besides, AOP have been reported as an alternative treatment technology for POME waste. This technology is based on the production of high and reactive hydroxyl (OH•) radicals to reduce organic pollutants [64]. AOP can be categorized as a photochemical or non-photochemical process that only relies on the processes, such as photochemical groups produced from direct photolysis by UV light, UV/TiO_2_, UV/H_2_O_2_, photo-Fenton, and photo-Fenton-like processes. In contrast, non-photochemical groups are produced by ozonation processes and Fenton [65]. Taha and Ibrahim [66] reported that OH• was successfully produced via the Fenton process to remove COD in POME waste. It was reported that the maximum COD reduction was 80% achieved within 2 h instead of 24 h of silent degradation after the sonification process, and there is no addition of oxidants. Organic decolorization and degradation in POME were also investigated using the Fenton process [67]. It was reported that the COD removal of 82% and color degradation of more than 90% was achieved by using 50 mM H_2_O_2_ with 1.0 mM Fe^2+^ for the POME oxidation process for 30 min.

Moreover, Saeed et al. reported that the Fenton oxidation process could degrade organic and inorganic compounds with a total COD removal of 85% under the acidic POME at an approximate pH of 3 [68]. Photocatalytic reactions show high efficiency in the mineralization of organic compounds and disinfection of pathogenic microorganisms in wastewater [69]. TiO_2_ is the most popular among the semiconductor catalysts because it has low toxicity, high chemical stability, high activity, and low cost [70]. Many studies have been conducted on photocatalytic use of TiO_2_ to degrade organic pollutants [71,72].

### 5.4. Integration Treatment

**Figure 12:**
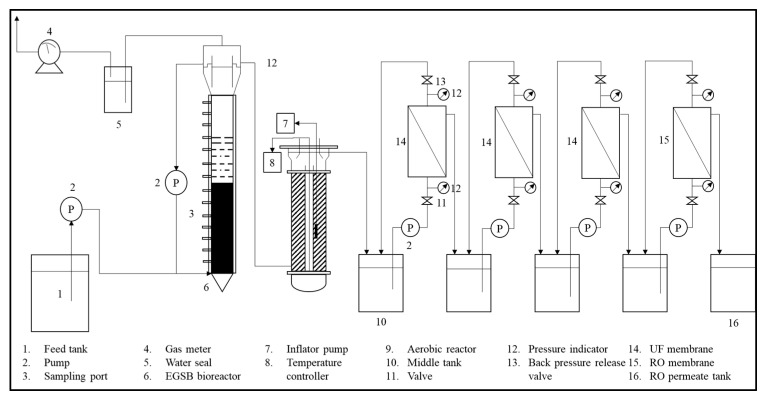
Schematic diagram of integration treatment for POME. Adapted with permission from ref. [60]. Copyright 2008 Elsevier.

Some treatments combine biological treatments with physical treatments, such as membrane technology [60]. The schematic diagram of the pilot plant for integration treatment is shown in Figure 12. This treatment’s initial stage is anaerobic and aerobic treatment using an EGSB reactor. About 43% of the organic material produced in POME is converted into biogas, while the efficiency of COD removal in anaerobic and aerobic reactors is 93% and 22%, respectively. In addition to COD and BOD removal, suspended solids and residual oil also decrease. In the membrane processing unit, almost all suspended solids are captured by the membrane. The effluent produced at the end of high-quality processing is very clear and can be used as boiler feedwater. However, the use of this treatment requires high costs, bearing in mind there are two stages of care needed. Table 3 summarizes comparisons of the technologies used for POME waste treatment.

## 6. Photocatalytic Technology for POME Treatment

### 6.1. Mechanisms and Fundamentals of Photocatalytic Technology

Photocatalytic technology is a combination of photochemical processes and catalysts. Materials that can be used as photocatalysts have an energy band gap like the oxides form of most transition metals. The bandgap is the energy between the conduction band and the valence band that produces a current carrier. The valence band is the energy level filled with electrons with a low energy state known as the highest occupied molecular orbital (HOMO). In contrast, the conduction band is an energy level that is not filled with electrons and is called the lowest unoccupied molecular orbitals (LUMO). Suppose the photocatalyst is subjected to light source energy equal to or greater than the energy bandgap. In that case, the light energy can promote electron excitation from the valence band to the conduction band, producing positive holes in the valence band. As a result of electrons’ transfer, conductivity is obtained and produces current when the electrode potential is sufficient [75,76].

Based on the type of catalyst, the photocatalysis process is divided into homogeneous photocatalysis and heterogeneous photocatalysis [77]. Homogeneous photocatalysis occurs in the same phase between reactants and photocatalysts (generally in the liquid phase). Homogeneous photocatalysts commonly used are hydrogen peroxide (H_2_O_2_), ozone, or other oxidants [78]. In contrast, heterogeneous photocatalysis occurs between two phases or more (generally, catalysts are present as solid phases). The most commonly used photocatalysts are semiconductors-based transition metals oxides (TiO_2_, ZnO, WO_3_, CeO_2_, ZrO_2_, etc.) [79,80,81,82,83].

**Figure 13:**
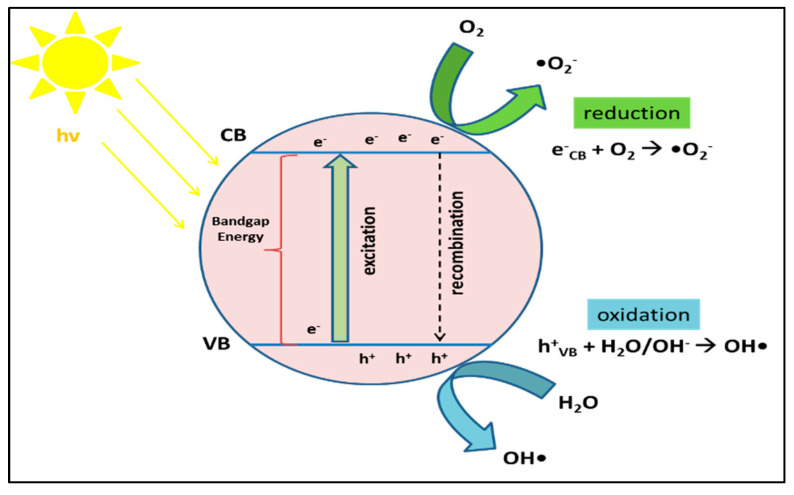
Schematic of photocatalytic mechanisms for organic pollutant degradation.

The schematic mechanism of the photocatalysis process can be illustrated in Figure 13. The heterogeneous photocatalysis process in semiconductor material begins with photoexcitation due to light hitting the semiconductor material. The light must have energy greater than or equal to the bandgap energy to transfer electrons from the valence band to the conduction band and produce a hole (h^+^) in the valence band called the electron–hole pair (Equation (1)). The redox process then occurs if there are compounds that are adsorbed on the surface of the semiconductor. A suitable scavenger will take this electron–hole pair to prevent the recombination process (Equation (2)). The electron in the conduction band will react with the electron acceptor, and the positive hole in the valence band will react with the electron donor. Electron acceptors (usually O_2_) will be reduced to other compounds during the electron transfer process, while electron donors will undergo an oxidation process. The reduction and oxidation process (redox) is utilized to suppress pollutants that contact the photocatalyst’s surface. Both electrons and holes can produce reactive radicals that can be used in the process of pollutant degradation. Electrons will interact with air or oxygen to produce superoxide radicals (•O_2_^−^) (Equation (3)), while holes interact with water molecules (H_2_O) to form hydroxyl radicals (OH•) (Equation (4)). Superoxide radicals can be converted to hydrogen peroxide (Equation (6)) through hydroperoxyl radical formation (Equation (5)). Hydrogen peroxide can then be converted to hydroxyl radical in the presence of light (UV/Visible) (Equation (7)). These radicals (hydroxyl radical, superoxide radical, and hydrogen peroxide) will degrade pollutant compounds into small molecules such as CO_2_, H_2_O, and mineral acids (Equation (8)).

Semiconductors + hv → e_CB_^−^ + h_VB_^+^ (1)

Semiconductors (e_CB_^−^ + h_VB_^+^) → Semiconductor + heat (2)

e_CB_^−^ + O_2_ → •O_2_^−^ (3)

h_VB_^+^ + H_2_O → OH• + H^+^ (4)

•O_2_^−^ + H^+^ → •OOH (5)

2•OOH → O_2_ + H_2_O_2_ (6)

H_2_O_2_ + hv → 2OH• (7)

pollutant + (OH•, •O_2_^−^) → CO_2_ + H_2_O (8)

The overall process that occurs in heterogeneous photocatalysis can be divided into five stages [69,84]: (i) diffusion of liquid-phase reactants to the surface of the catalyst; (ii) adsorption of reactants onto the surface of the catalyst activated by photons; (iii) photocatalyst reaction in the adsorbed phase on the catalyst surface; (iv) desorption of substances/products from the surface of the catalyst; (v) separation (transfer of mass of substance/product from the interface area).

### 6.2. Development of Photocatalytic Process for POME Treatment

#### 6.2.1. Semiconductor Based Photocatalyst

##### TiO_2_ Photocatalyst

**Figure 14:**
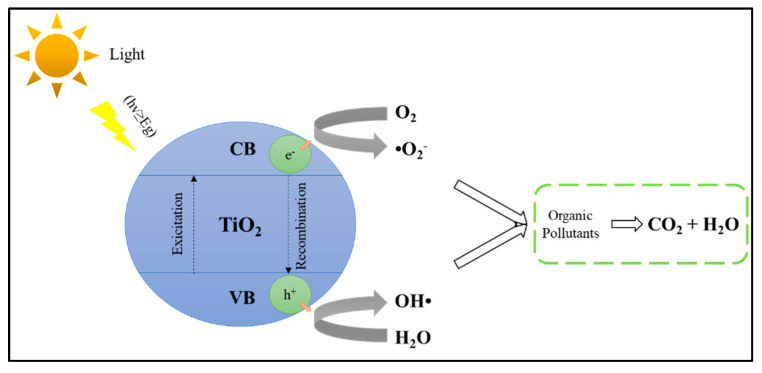
The schematic mechanism of photocatalysis using a semiconductor-based catalyst.

Titanium dioxide (TiO_2_) is a natural oxide from the element titanium and is known as titania. TiO_2_ has several advantages, including cheap, non-toxic, good photocatalytic activity, abundant availability, wide bandgap, insoluble in water, high thermal and chemical stability, and has a large surface area [85,86,87,88,89,90]. TiO_2_ has three types of crystalline forms, namely anatase, rutile, and brookite [90]. Currently, TiO_2_ has been studied extensively in physiochemical, toxicological, and biocompatibility studies [91]. In all three forms, the commonly used forms are anatase and rutile. However, the anatase form has excellent physical and chemical properties in waste treatment and is thermodynamically more stable than the rutile phase [92]. For TiO_2_, rutile and anatase forms have a high bandgap of 3.0 eV and 3.2 eV, respectively [93]. Therefore, to activate TiO_2_, high-energy UV light radiation is needed with a wavelength of not more than 387.5 nm [94]. Due to the abundance of UV light in nature, it is necessary to make an effort to make TiO_2_ efficient as a photocatalyst in waste treatment. The electron–hole pair on TiO_2_ tends to be easy to recombine (recombination) and has a relatively low adsorption capacity [95,96]. Figure 14 shows the general scheme of reactions that occur when using the semiconductor-based catalyst for POME degradation.

##### WO_3_ Photocatalyst

Tungsten trioxide (WO_3_) is a yellow chemical compound containing oxygen and tungsten transition metals. WO_3_ is often used for everyday purposes as a pigment in ceramics and paints. WO_3_ crystal structure varies depending on temperature; at room temperature will be monoclinic. WO_3_ has several advantages, including a semiconductor with a narrow bandgap, has good photocatalyst activity in non-toxic acidic solutions environmentally friendly, has strong adsorption power, and high thermal and physicochemical stability [97,98,99,100,101,102]. WO_3_ has a bandgap between 2.7 and 2.8 eV when compared with TiO_2_ 3.0–3.2 eV. It can absorb UV light until the visible light in a greater solar spectrum and have a better visible light absorbance photo [103]. Since the absorption spectrum of WO_3_ in the range of UV light and visible light, WO_3_ has the potential of being a visible photocatalyst [97,100,104]. However, these materials are scarce and thus it is expensive (varies by country) [105]. Besides, pure WO_3_ has a small surface area, and the high level of electron–hole recombination makes WO_3_ photoactivity low [101,105]. Cheng et al. [106] evaluate the photocatalytic treatment of POME waste over tungsten oxide photocatalyst (WO_3_) with UV irradiation. At optimal catalyst loading (0.5 g/L) produced the highest photocatalytic degradation (51.15%) and decolorization (96.21%) within 1 h of treatment. For longevity study of WO_3_, the optimum reaction time was 16 h, reaching 84.70% photocatalytic degradation and 98.28% photocatalytic decolorization.

##### ZnO Photocatalyst

Zinc oxide (ZnO) is an inorganic compound that is not soluble in water in white powder and is widely used as an additive in various materials. ZnO has two crystal structure structures, known as hexagonal wurtzite and cubic zincblende. The commonly used ZnO is in the form of wurtzite because of its high stability at room temperature [107].

ZnO has a wide bandgap of 3.2 eV that is the same as TiO_2_; therefore, it is estimated that its photocatalytic ability is similar to TiO_2_ [108]. ZnO is an environmentally friendly material [109]. ZnO also has important properties such as the extreme stability of excitons (indicated by the large exciton binding energy) and the large bond strength is indicated by the melting point and cohesive energy) [110]. ZnO is also relatively cheaper than TiO_2_ because TiO_2_ is quite wasteful for large-scale water treatment [111]. Another advantage of TiO_2_ is that it can absorb the UV spectrum fraction in the solar that is greater, and the appropriate ZnO threshold is 425 nm [112]. This view is supported by research that TiO_2_ can only absorb 3% of UV light from the solar spectrum and has a low photocatalyst efficiency [113]. ZnO’s weakness has a wide bandgap like TiO_2_, but ZnO is a photocorrosion material. A wide bandgap causes the limitation of absorption of light in the visible light region. This condition can cause low photocatalytic efficiency and result in rapid recombination [107]. Study by Ng et al. [114] reported that photocatalytic methods have been used to restore POME waste with ZnO photocatalysts with UV irradiation. The degradation process increases consistently with photocatalyst loading until the optimal point is reached at the 1.0 g/L photocatalyst. Under these conditions, the ZnO system achieved a degradation of 49.36%. Beyond 1.0 g/L, degradation has slightly decreased with photocatalyst loading due to the effect of light scattering from excess photocatalysts. Besides, a long-life study (22 h) showed a degradation of 74.12% for the ZnO system.

There are still many reports from previous photocatalytic works in the open literature using different photocatalysts summarized in Table 4. Different photocatalysts will show different results in POME waste due to the nature of both. Although, activators (UV light/visible light) also play a very important role in photocatalysis. For example, a material with a wide bandgap can only be activated by UV light. Therefore, the selection of photocatalysts and activators is very important to ensure the effectiveness of organic degradation.

#### 6.2.2. Modification and Doping of the Semiconductor Based Photocatalyst

The energy band in semiconductor photocatalyst is an important factor in photocatalytic reactions. The range of light energy that photocatalysts can absorb depends on the energy bandgap. The wider the bandgap of energy it will limit its use in visible light. As explained in Section 6.1, the basic principle of photocatalysis depends on electron–hole excitation. The electrons in the valence band can be excited into the conduction band, and photon energy stimulation is needed. A wider bandgap of energy requires more photon energy to excite the electron–hole. For example, the anatase type TiO_2_ band gap is 3.2 eV [128], which shows that electrons can only be excited by light with more energy, that is, UV light.

Of the various existing photocatalysts, TiO_2_ has become a photocatalyst that has received much attention. Many researchers are making efforts to overcome the weakness of TiO_2_ photocatalyst, which has been described in Section 6.2.1. These efforts are modifying TiO_2_ photocatalysts to have a narrower energy band, a slow recombination rate, accelerating interfacial charge transfer. All these efforts aim to get better photocatalytic activity. One method of photocatalyst modification is through doping. It can control the semiconductor’s bandgap structure by adding a small number of impurity atoms (dopants). This section explains methods for modifying TiO_2_ by doping methods and their effects on photocatalytic activity.

##### Doping

**Figure 15:**
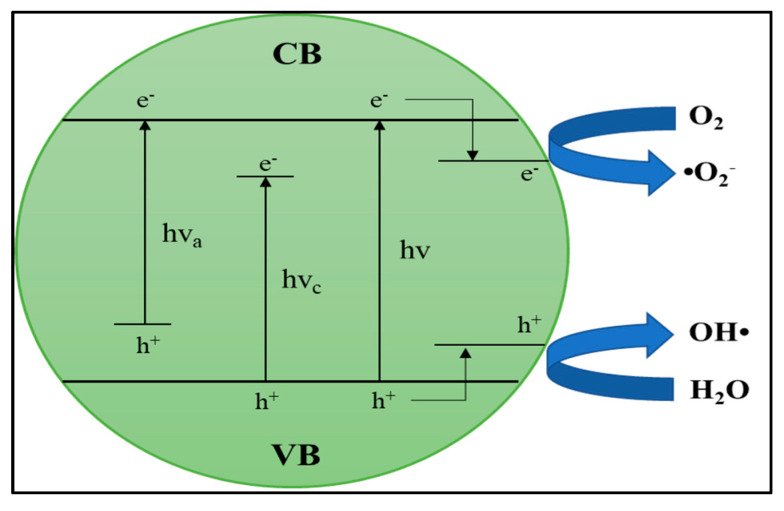
Simplified pure and doped TiO_2_ photocatalyst mechanism: doping reduces bandgap, facilitating photoexcitation and reactive radical production. hv: pure TiO_2_; hv_c_: cation-doped TiO_2_; hv_a_: anion-doped TiO_2_.

In addition to the bandgap parameter, the charge is carried by electrons and holes in photocatalysts, carrying negative and positive electrical charges. Pairs of electron holes are created in the photocatalyst’s outer surface region when exposed to light (photon energy). However, electron–hole pairs also tend to rejoin and recombine. However, a high charge carrier mobility and a long charge carrier diffusion length are needed to achieve the low level of electron recombination needed for photocatalytic activity. A charge carrier trap is needed to reduce the rate of electron–hole recombination. In addition to narrowing the bandgap energy, doping can act as a charge carrier trap to produce good photocatalytic activity (Figure 15).

##### Cation Dopants

**Figure 16:**
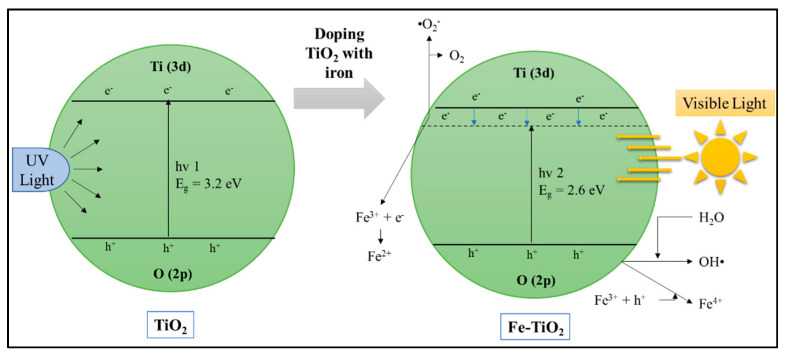
The schematic energy level of undoped TiO_2_ and iron-doped TiO_2_ (Fe-TiO_2_).

Photocatalysts can be modified with dopant cations, and dopant cations consist of transition metals, noble metals, and rare earth metals. This modification aims to make photocatalysts absorb visible light. Transition metals such as Cu [116], Fe and Cu [129], Co [130], Ni [131], Mn [132], Zn [133], etc., have been widely studied for doping photocatalysts. Photocatalyst doping with transition metals can change electronic structures that cause UV light absorption changes in visible light [134]. This condition increases photocatalytic activity—for example, photocatalyst TiO_2_, which has Ti 3d and O 2p atomic orbitals. If doped with Fe, which has 3d atomic orbitals, it can shift the CB boundary and narrow the photocatalyst energy bandgap, as presented in Figure 16. If the bandgap energy is narrower, it can shift absorption into visible light as the photocatalyst’s doped metal concentration increases. Increased absorption in visible light may be due to the transfer of electrons from the metal ion orbitals to the CB photocatalyst. For instances, Ng et al. [116] reported that 20 wt % Cu/TiO_2_ exhibited two-folds enhancement of photocatalytic rate constant for POME degradation compared to 2 wt % Cu/TiO_2_, which was due to larger pore diameter [116]. In addition, doping TiO_2_ with 0.5 wt % of Ag (0.5 wt % Ag/TiO_2_) exhibited 3.5 and 8.6 times higher photocatalytic rate constant for POME degradation under UV and visible light illumination, respectively. This enhancement can be attributed to the narrower band gap energy of Ag/TiO_2_ and thus improved visible light absorption. In addition, Ag may have also enhanced the charge separation by rapidly-transferring the e^−^ away from the positive h^+^ charge on the TiO_2_ surface, thus minimizing charge recombination [121].

Metal with redox potential can act as an electron trap to function as an electron acceptor to hold electron–hole pairs’ recombination. On the other hand, electrons trapped in metals that have a high reduction potential can cause a reduction of some metals and consume electrons instead of moving them to the surface [135]. Transition metals with two or more oxidation states, such as Fe and Ce, with different ionic forms (Fe^2+^, Fe^3+^, and Fe^4+^ and Ce^3+^ and Ce^4+^) can also act electron–hole traps and inhibit the recombination of electron–hole pairs. However, electron holes’ recombination rate can increase with increasing metal concentrations because metal ions acting as electron traps and hole traps will then form various trap sites. Suppose there are many charge traps in the bulk of catalysts or on the surface surfaces. In that case, the mobility will be low, and possible to recombine electron–hole pairs before reaching the surface [136].

##### Anion Dopants

**Figure 17:**
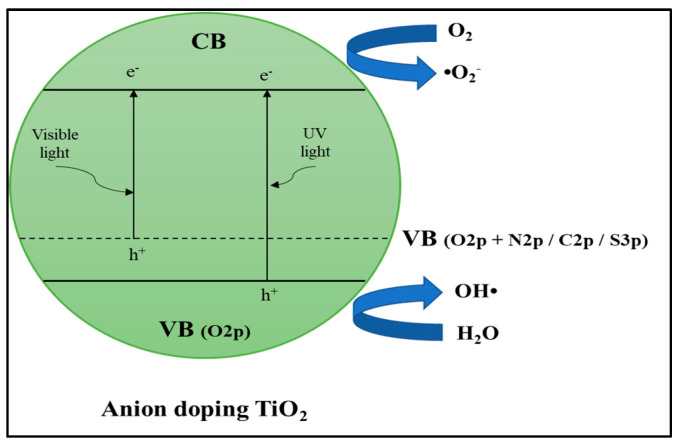
The schematic energy level of anion modified TiO_2_ photocatalyst.

TiO_2_ can also be doped on O sites with anions such as nitrogen (N) [137], sulphur (S) [138], carbon (C) [139], and boron (B) [140]. The combination of p orbitals from dopant anions (N, S, C, and B) with O 2p orbitals increased the valence band (VB) and can narrow the photocatalyst energy bandgap (Figure 17). Doping using nonmetallic carbon (C) can also be an electron trap from electrons produced by photoexcitation, reducing electron–hole pairs’ recombination rate. Carbon (C) has a wide absorption spectrum area of 400–800 nm to encourage a charge transfer from the inside of the photocatalyst to the surface. Carbon doped TiO_2_ showed significant changes from the absorption edge with calcination temperatures of 200, 300, 400, and 500 °C having absorption edges at 390, 400, 410, and 450 nm. The absorption shifted slightly towards the visible region compared to commercial TiO_2_ at 385 nm [141]. Kalantari et al. [142] reported that the energy of N doped TiO_2_ band gap of 2.76 eV is lower than TiO_2_-P25 of 3.1 eV. Nitrogen dopants, which are incorporated into the TiO_2_ framework, reduce the energy gap of the TiO_2_ band, and increase the absorption of visible light. This phenomenon can be related to either the formation of N energy levels above the valence band of TiO_2_ or the mixing of nitrogen and oxygen states. Ananpattarachai et al. [143] found that N-doped acts to prevent the recombination of electron–hole pairs produced by photoexcitation, at high temperatures, N-doped TiO_2_ was prepared by thermal treatment of commercial TiO_2_ with NH_3_ gas flow. Jo et al. [138] reported that the bandgap energy of S-TiO_2_ is 2.75 eV. The absorption spectrum of S-TiO_2_ considerably shifted towards the visible region. These shifts were attributed to increased charge transfer rates between S and TiO_2_ because it is impregnated and/or replaces S atoms in the TiO_2_ lattice, producing impurity levels that can reduce the gap of the TiO_2_ band. This condition suggests that the prepared S-TiO_2_ can function effectively under visible light irradiation. Thus, anion dopants on semiconductor-based photocatalyst can be further investigated to enhance photocatalytic performance of POME degradation.

##### Anion–Anion Dopants

**Figure 18:**
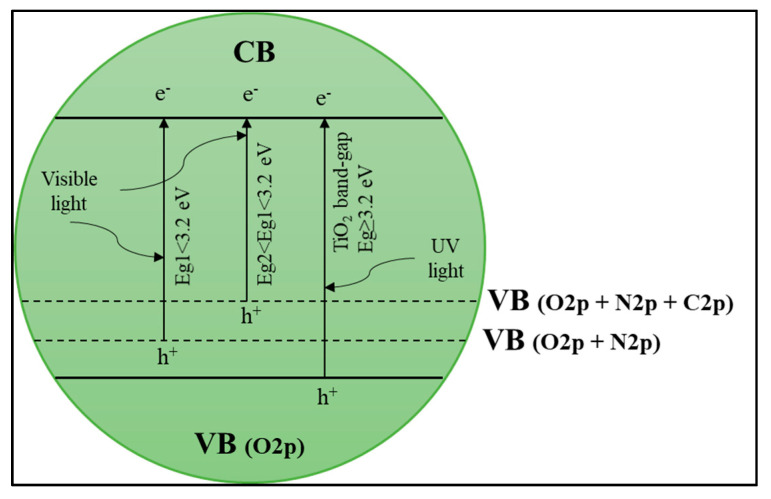
Schematic of new energy bands formation in C–N–TiO_2_ photocatalyst.

The purpose of codoping TiO_2_ with different elements to increase the photocatalytic activity of TiO_2_ and the more effective use of solar light in the visible light region has received much attention photocatalytic field. As mentioned in the previous section in the bandgap narrowing problem, anion dopants are more efficient than cation dopants. Therefore, reports on TiO_2_ doped with non-metals and non-metals will be reviewed in this section. Zhang et al. [144] reported that non-metal and non-metal doped TiO_2_, the energy levels of 2p orbitals each contribute to creating new energy states in the TiO_2_ bandgap synergistically, as observed in codoping with C and N (Figure 18). In the VB TiO_2_ state, there is an overlap between C 1s and N 1s facilitated by the level of C-doping energy connected to the N-doping state [145]. Moreover, Komai et al. [146] also found high photocatalytic activity due to energy bandgap narrowed on N and S codoped TiO_2_. It was reported that visible light’s photocatalytic activity is better by the synergistic effect of doping C and B. Boron doping effectively narrows the bandgap of TiO_2_ while doping C produces carbon coke, which can act as photosensitizers [147]. Thus, anion–anion dopants on semiconductor-based photocatalyst can be further studied to improve photocatalytic performance of POME degradation.

##### Cation–Cation Dopants

**Figure 19:**
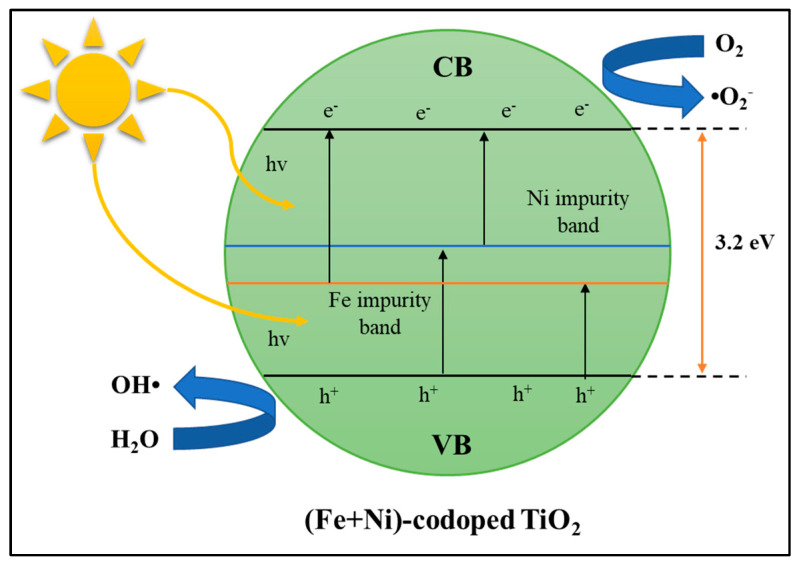
Schematic illustration of energy band level in (Fe + Ni)-codoped TiO_2_ system.

It has been widely reported that TiO_2_ doped with the right elements can show better photocatalytic activity than pure photocatalysts. There is a synergy between TiO_2_ doped metals resulting in effective performance [135]. (Fe, Ni) codoped TiO_2_ nanoparticles were successfully prepared by the alcohol-thermal method by Sun et al. [148]. The edge light absorption of Fe-Ni/TiO_2_ moves remarkably with a redshift to the visible range. Fe and Ni doping can produce impurities in the crystal lattice of TiO_2_, and that band is located in the middle of the optical bandgap of TiO_2_. Electrons in the valence band absorb photons with longer wavelengths, and firstly transfer to the impurity band (a relatively higher energy state), then secondly transfer from the impurity band to the conduction band through absorbing other photons (Figure 19). Therefore, the optical absorption of metal-doped samples depends on the impurity band in the TiO_2_ lattice. The Fe and Ni codoping display a higher optical absorption of visible light than single doping. Due to Fe and Ni’s codoping, the intensity of the absorption of visible light of TiO_2_ increases, which is an important cause of higher photocatalytic activity under visible light irradiation. The energy bandgap from Fe-Ni/TiO_2_ is estimated from 2.41 to 2.56 eV, depending on the Fe/Ni ratio. Talat-Mehrabad et al. [149] reported that TiO_2_ photocatalysts doped with Ag-Mg prepared by the photodeposition and impregnation methods had a narrower energy band than single doped photocatalysts. Besides, the TiO_2_ Ag-Mg photocatalyst absorption band also appears to be shifting toward the visible light region. The rate of recombination of electron–hole pairs is slower than single doped TiO_2_. Considering the advantages of cation–cation dopants compared with neat semiconductor based photocatalyst, it will be worth to further studied the effect of cation–cation dopants on semiconductor based for photocatalytic POME degradation.

##### Cation–Anion Dopants

As discussed in the previous section, anion dopants can narrow the bandgap energy better than cation dopants, but anion dopants tend to form the center of recombination. While cation dopants have excellent performance in reducing electron recombination pairing, metal ions suffer from thermal stability problems. Therefore, codoping cations and anions on TiO_2_ are considered to overcome the weaknesses in doping TiO_2_ with single metals and non-metals. The electronic structure of TiO_2_ will change the effect of metal and non-metal ions by creating new doping levels in the bandgap [135]. The synergistic effect of doping between metals and non-metals will increase the excitation rate of electrons and holes and increase the photocatalytic activity of TiO_2_ in the visible light region.

**Figure 20:**
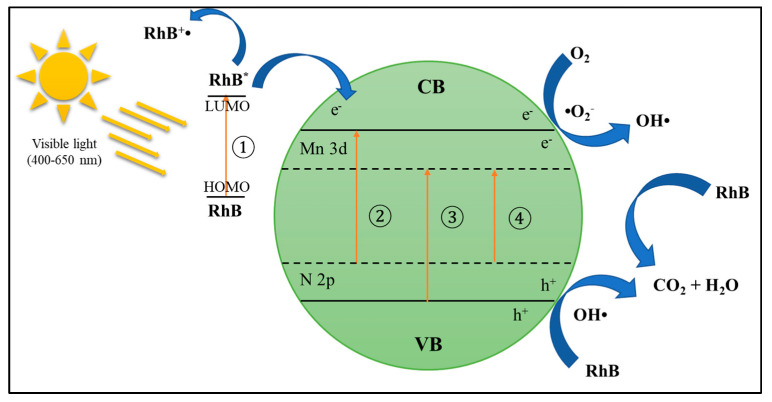
Schematic illustration for photocatalytic degradation of RhB over Mn-N-codoped TiO_2_ under visible light irradiation.

Quan et al. [150] reported that Mn-doped TiO_2_ showed significant photocatalytic activity under irradiation of visible light compared to pure TiO_2_, and codoping Mn and N further enhanced this activity into TiO_2_. The Mn doping could narrow the TiO_2_ bandgap extending the absorption range of TiO_2_ to visible light and inhibits the recombination of electrons and photogeneration holes, which leads to a better increase in photocatalytic activity in the visible light region. Additionally, Mn-N-TiO_2_ shows the absorption of visible light stronger than Mn-TiO_2_. The relatively strong absorption at 400–650 nm was attributed to the codoping of N and Mn elements into the lattice of TiO_2_. It is generally accepted that doping N can form a narrow N 2p band isolated above the O 2p TiO_2_ valence band, reducing the gap of the TiO_2_ band and absorbing visible light. As illustrated in Figure 20, Mn and N ions’ synergistic effect narrows the bandgap of TiO_2_, which forms a new closed state, respectively, in the conduction band and valence band.

Jaiswal et al. [151] reported that TiO_2_ doped with V and N had better visible light absorption efficiency than single V and N doped TiO_2_. This condition is caused by narrowing the bandgap effect of the simultaneous merging between V and N into TiO_2_. Besides, it was reported that La_3_^+^ doping could withstand the recombination rate of electron–hole pairs. In contrast, doping N could reduce the TiO_2_ bandgap and increase the efficiency of TiO_2_ absorption in the visible light region [152]. Gaikwad et al. [153] reported that codoping TiO_2_ with Fe and N narrowed the bandgap of TiO_2_ and showed increased absorption of visible light and showed increased photocatalytic activity. It was found that M, N codoped TiO_2_ specimens have higher photocatalytic capabilities than pure TiO_2_ and mono-doped TiO_2_ under visible light irradiation. Besides, bandgap and carrier mobility in VB, CB, and impurity levels (ILs) have a synergistic effect on the absorption of visible light and photocatalytic activity of doped TiO_2_. The impurity states between VB and CB increase the absorption of visible light. The concentration of N in the codoped specimen effectively affects IL states. The amount and mobility of IL carriers together influence the photocatalytic activity of the catalyst under visible light. Thus, Mn, N codoping specimens showed better photocatalytic activity [154]. Considering the improvement of photocatalytic performance for organic degradation (i.e., rhodamine blue) by adding cation–anion dopants on semiconductor, it is highly recommended to further observed the effect of cation–anion dopants on photocatalytic POME degradation.

##### Other Semiconductors Dopants

Combining TiO_2_ photocatalysts into hybrid forms with other semiconductors with narrow bandgaps such as CdS, Cu_2_O, Bi_2_S_3_, and SnO_2_ are other strategies for modifying energy bands, inhibiting the rate of recombination of electron–hole pairs to increase photocatalytic activity. The principle of dopants with other semiconductors is that narrow bandgap semiconductors absorb photon energy from visible light. Photogeneration electrons are then transferred from the narrow bandgap semiconductor CB to the CB TiO_2_. Electrons can only be transferred if the narrow band edge semiconductor CB edge is more negative than the CB TiO_2_ edge [135]. Bessekhouad et al. [155] reported that the higher the CB difference between two semiconductors, the higher the electron transfer driving force. CdS nanoparticles not only act as sensitizers but also reduce the rate of photogenerated charge carrier recombination. Besides, the results of photoactivity showed that TiO_2_ doped by CdS exhibited better photocatalytic performance [156]. Boumaza et al. [157] also reported that the azo Orange G dye was successfully degraded in the hetero-system x% Bi_2_S_3_/TiO_2_ under visible light. Loading TiO_2_ with Bi_2_S_3_ greatly enhances photoactivity due to the transfer of electrons from Bi_2_S_3_ to TiO_2_ by the synergy effect. This increased photo activity is caused by Bi_2_S_3_ dispersion, which effectively increases the reception of visible photons. SnO_2_ surface coupling to TiO_2_ acts as a trap for photogeneration electrons. It thereby decreases the rate of recombination of electron–hole pairs and further increases the photocatalytic activity of TiO_2_ under irradiation of visible light [158]. Cu_2_O@TiO_2_ nanoparticles showed increased photocatalytic degradation when compared to pure Cu_2_O nanocubes and TiO_2_ nanoparticles. The consequence of photoelectrochemical measurements shows that the composite heterojunction of p-Cu_2_O/n-TiO_2_ can facilitate the transfer of electrons across the heterojunction interface, advantageous for improving photocatalytic performance. The experimental results show that Cu_2_O nanocubes extensively enhance the TiO_2_ response, which shows higher activity compared to neat TiO_2_ [159]. In the case of POME treatment, Chin et al. [127] reported that 3 wt % Nb_2_O_5_/ZnO exhibited 3.7 and 1.4-folds enhancement of COD removal after 240 min and color removal after 30 min photocatalytic reaction durations, respectively. Taking into account the advantages of heterojunction modification strategies, it is very worthy to further study the effect of heterojunction of two semiconductors on the photocatalytic performance of POME degradation.

### 6.3. Post-Processing Recovery of Photocatalyst for POME Treatment

Photocatalyst recovery and separation from POME waste is an essential step for catalyst recycling and releasing the degraded POME waste. The way to immobilize or separate photocatalyst particles effectively in the photocatalytic process remains a challenge. In general, in order to solve recovery and separation issues, two potential approaches have been investigated namely magnetic separation and immobilization on support structures. Utilization of magnetic separation provides facile and convenient approach for recovering and separating photocatalyst particles. A number of materials with different elemental compositions, such as NiFe_2_O_4_ [160], CoFe_2_O_4_ [161], Co_3_O_4_ [162], γ-Fe_2_O_3_ [163], and Fe_3_O_4_ [164], have been obtained as the magnetic cores. Among all these magnetic materials, Fe_3_O_4_ is the most widely used due to its low toxicity, biocompatibility, and excellent magnetic properties [164]. On the other hand, immobilization photocatalyst on various supports, such as glass, quartz, stainless steel, and fibers have been also studied [165,166]. This approach provides a facile way to solve separation and aggregation issues. However, this approach could reduce active surface area and volume ratio, decrease mass transfer rate, and hindrance in light absorption, thus photochemical reactivity becomes the main issues.

Therefore, few studies concerned on the development of other approaches to recover and separate photocatalyst materials have been conducted including: (1) alkaline treatment (NaOH and NH_4_OH) [167], (2) thermal regeneration [168], (3) exposure to UV in aqueous media [169], (4) oxidation by H_2_O_2_/UV [167], (5) washing with deionized water [170], (6) refluxing in water at 100 °C with oxygen bubbling [171], (6) chemical coagulant (aluminum chloride [172], chitosan [173], and ferric chloride [174]), and (7) ceramic membrane microfiltration [175]. Miranda-Garcia et al. [167] reported that thermal and H_2_O_2_/UV are more efficient recovery strategy compared to alkaline treatment due to TiO_2_ was partially removed by alkaline treatment leading to the decrease of photocatalyst’s performance. Cui et al. [175] reported that ceramic membrane microfiltration could efficiently recover TiO_2_ photocatalyst in a slurry reactor by achieving 99.9% recovery rate, realizing a continuous operation for wastewater treatment. This post-processing recovery strategies option can be further studied with respect to the application of photocatalytic POME degradation both in lab scale and pilot scale applications.

## 7. Operational Parameters/Factors Affecting the Photocatalytic Degradation Process

In addition to the previously discussed factors of photocatalysts, effective POME waste treatment or the photocatalytic system’s efficiency is greatly influenced by several operating parameters or factors that control the photocatalytic kinetics. This section will discuss several of these operating parameters that affect the photocatalytic activity and the performance of TiO_2_ photocatalysts in POME waste treatment.

### 7.1. Catalyst Loading

The concentration of TiO_2_ in the photocatalytic system in POME waste treatment affects the rate of heterogeneous photocatalytic reactions. The concentration of TiO_2_ directly affects the rate of photocatalytic reactions [176]. Initially, the effect of TiO_2_ concentration is linear to some extent. However, when the concentration of TiO_2_ increases above the saturation limit (different concentration of TiO_2_ causes turbidity of the solution), there will be a corresponding radial decrease in the coefficient of light absorption (photon energy) and subsequently causes a decrease in the surface area exposed to light irradiation and will reduce the efficiency of the photocatalytic process. Therefore, each photocatalytic process must be operated below the saturation level of the TiO_2_ concentration to avoid excess photocatalysts and ensure efficient absorption of light (photon energy) [69]. Several studies were conducted to examine TiO_2_ concentration on process efficiency [176,177]. However, the effect cannot be found, and a direct connection cannot be made. Additionally, it is reported that optimal photocatalyst loading for photomineralization and photodisinfection can vary [69]. Based on the Table 4, it can be seen that the optimum catalyst loading for photocatalytic POME treatment is in the interval between 0.1 and 1.5 g/L.

### 7.2. pH

One important parameter in a heterogeneous photocatalytic system is pH. These parameters determine the nature of the charge on the photocatalyst’s surface, the photocatalyst’s aggregate size, the conduction band’s position, and the valence [69]. Many attempts to research and study the effect of pH on photocatalytic activity, one of which uses the point of zero charges (PZC) of TiO_2_. PZC is a pH value where the surface charge component is equal to zero under given conditions of temperature, applied pressure, and soil solution composition [159] (PZC = 6–8 depending on TiO_2_ sample) [178]. Suppose pH < (PZC)TiO_2_, the photocatalyst’s surface charge is positive and is gradually given electrons by organic compounds adsorbed to the TiO_2_-activated photon to undergo different photocatalytic reactions. Suppose pH > (PZC)TiO_2_, the surface of the catalyst will be negatively charged and reject anions in water [69]. Based on the water equilibrium equation, the following reaction equation is obtained:

At pH < PZC: TiOH + H^+^ ⇌ TiOH_2_^+^ (9)

At pH > PZC: TiOH + OH^−^ ⇌ TiO^−^ + H_2_O (10)

### 7.3. Temperature

Chong et al. and Gaya et al. [69,176] stated that an increase in temperature in the photocatalytic reaction (>80 °C) would cause an increase in the recombination of electron–hole pairs and inhibit the adsorption of organic compounds on the TiO_2_ surface leading to a decrease in photocatalytic activity. This statement is in accordance with the Arrhenius equation, where Kapp’s clear first-order rate constant must increase linearly with exp (−1/T). Conversely, at temperatures below 80 °C, adsorption is an exothermic event that occurs spontaneously, and the adsorption of the final reaction product will increase. This finding is also supported by Malato et al. [179], which in the temperature range of 20–80 °C it has activation energy that is often very small (several kJ/mol) where the activation energy is zero. However, the activation energy at temperatures below 0 °C increases. Furthermore, desorption of the final product becomes rate-limiting. Therefore, the optimum temperature generally consists of between 20 and 80 °C.

### 7.4. Size and Structure of the Photocatalyst

Photocatalytic activity is also influenced by the structure and size of the crystals, especially in the form of nano. For example, TiO_2_ structurally has three crystalline phases: anatase, rutile, and brookite. However, among these three types of structures, only anatase and rutile are quite stable. Different types of structures certainly affect the difference in density (3.9 g/cc for anatase and 4.2 g/cc for rutile), and of course, this can affect the surface area and active side of the TiO_2_. In addition, the crystal structure turns out to result in differences in the energy level of the electronic band structure (bandgap energy). The amount of bandgap energy (E_g_) between anatase and rutile will differ if Ti and O atoms’ arrangement in TiO_2_ crystals is different. The anatase structure has an energy gap of 3.2 eV and rutile has an energy gap of 3.0 eV [93].

Saquib et al. [180] shows that photocatalytic activity also depends on the type of pollutant model. In the study, it was found that Degussa P-25 TiO_2_ showed better photocatalytic activity for degradation of Acid Orange 8 dyes and a large number of organic compounds than other TiO_2_ catalysts, namely Hombikat UV100 (100% anatase) and PC500 (100% anatase, 100% inorganic chemicals (Millennium)). This finding can be explained by the fact that Degussa P25 is a mixture of 25% rutile and 75% anatase. This research result is supported by Ohno et al. [181] and Muggli et al. [182], that the mixture of anatase (70–75%) and rutile (30–25%) is more active than pure anatase. On the other hand, the UV 100 Hombikat photocatalyst was better for the degradation of benzidine and 1,2-diphenylhydrazine, as shown in a recent study reported by Muneer et al. [183].

The size of the photocatalyst crystal also plays an important role in photocatalytic efficiency. Ma et al. [184] showed that doping inhibited the transformation of the anatase phase into rutile and inhibited the growth of crystallites. In addition, doping can expand the absorption area to the visible light region. Crystal size can be calculated using the Scherrer equation as shown in Equation (11):

(11) $$L=\frac{K\;\lambda }{\beta \;\cos\theta }$$

where λ is the wavelength of X-rays in nanometers (nm), β is the peak width of the diffraction peak profile at half the maximum height resulting from the size of small crystals in radians, and K is the constant associated with the shape of the crystal, usually taken as 0.9 [185].

### 7.5. Dissolved Oxygen (DO)

Dissolved oxygen (DO) has an essential role in the photocatalytic reaction. It is well known that DO can act as an electron acceptor to eliminate photogeneration recombination of electron–hole pairs and photocatalysts with better electron and hole separation. This condition allows more efficient channeling of charge carriers into useful reduction and oxidation reactions [186]. Moreover, the existence of oxygen could facilitate the formation of hydroxyl and superoxide radicals which act as reactive species for POME degradation.

Gerischer et al. [187] reported that suppose molecular oxygen is used as an electron acceptor to trap and remove electrons from a titanium particle’s surface to minimize free-electron buildup. The oxygen reaction adsorbed with photogeneration electrons on the titanium catalyst surface is relatively slow and maybe a step in controlling the rate of photocatalytic oxidation reactions.

Therefore, increasing the rate of charge transfer from titanium to molecular oxygen will increase photocatalytic efficiency for photo-oxidation of organic substrates. If the oxygen absorbed exceeds the electrons’ photogeneration on the surface, the electron transfer rate to molecular oxygen will be maximized. However, titanium’s type and characteristics are influenced by electron–hole generation efficiency, recombination, and the charge of transfer reaction rates [188].

### 7.6. Light Wavelength

The photochemical effect of light sources with different wavelength emissions will have considerable consequences on the rate of photocatalytic reactions depending on the type of photocatalyst used (crystalline phase and anatase–rutile composition). For example, Degussa P-25 TiO_2_ has an anatase crystal ratio of 70/80: 20/30, and the wavelength of the light is less than 380 nm enough for photonic activation [84,189]. The TiO_2_ rutile crystalline phase has a smaller energy bandgap of around 3.02 eV than the TiO_2_ anatase of 3.2 eV [93]. Therefore, rutile TiO_2_ can be activated with wavelengths of light up to 400 nm, depending on the bandgap threshold for the type of rutile TiO_2_ used.

The electromagnetic spectrum can be classified into UV-A, UV-B, and UV-C according to the wavelength emitted for UV radiation. UV-A has a wavelength range between 315 and 400 nm (3.10–3.94 eV), while UV-B has a wavelength range of 280–315 nm (3.94–4.43 eV), and UV-C has a wavelength range of 100–280 nm (4.43–12.4 eV) [190].

### 7.7. Light Intensity

Light intensity is one of several parameters that affect the rate of photocatalytic reactions for organic compounds degradation. Fujishima et al. [191] shows that photocatalytic reactions are not too dependent on the intensity of light, where some photons have only enough energy to induce reactions on the surface. To achieve high photocatalytic reaction rates, especially in wastewater treatment, relatively high light intensities are needed to adequately cover each active side of TiO_2_ with the required energy.

Ollis et al. [192] show that the effects of light intensity on photocatalytic efficiency can be categorized into three groups: (1) at low light intensities (0–20 mW/cm^2^), the rate of increase in linear reactions with an increase in light intensity due to the formation of more dominant electron holes and recombination of electron holes is ignored; (2) at medium light intensity (about 25 mW/cm^2^), the rate of reaction depends on the square root of the light intensity because at this stage, the electron–hole and recombination holes compete; (3) at high light intensity, the rate of reaction does not depend on the intensity of the light. With increasing light intensity, the number of activation sites remains the same so that the reaction rate only reaches a certain level even when the light intensity continues to increase.

This finding is supported by Reutergådh et al. [193] showing that the reaction rate increases around 2.2 times when the light intensity doubles. Under higher lighting intensity, the increase of reaction rate is much lower. This condition may result from the fact that low-intensity reactions involving the formation of dominant electron–hole pairs and recombination of electron–hole pairs can be ignored. However, on increasing the intensity of light, the separation of electron pairs competes with recombination, causing a smaller effect on the reaction rate. For TiO_2_ photocatalysis, the relationship of light intensity versus reaction rate is near linear. The intensity of UV light applied in the experiment (0–20 mW/cm^2^) corresponds to weak lighting. Based on the Table 4, it can be seen that by varying light intensity and wavelength, it affects the degradation rate (i.e., COD and BOD removal and decolorization) of POME waste.

## 8. Kinetic of Photocatalytic POME Degradation

It is widely believed that the kinetics of the photocatalytic reaction following the Langmuir–Hinshelwood (L–H) equation is seen in the following formula:

(12) $$r=-\frac{\mathrm{dC}}{\mathrm{dt}}=\frac{k\cdot K\cdot C}{1+K\cdot C}$$

where k is the reaction rate constant, K is the reactant adsorption constant, and C is the reactant concentration each time. To calculate the reaction rate in a heterogeneous system, it takes the value of the reactant adsorption constant (K) on the catalyst’s surface. Since no experiments were carried out to calculate the K value in this study, the calculations were carried out with a homogeneous system approach.

The equation for the reaction rate is:

(13) $$r=-\frac{\mathrm{dC}}{\mathrm{dt}}=k\cdot C^{n}$$

where k is the reaction rate constant and n is the reaction order. The reaction order and reaction rate constants are determined by integrating the reaction rate equation into a linear equation.

**Figure 21:**
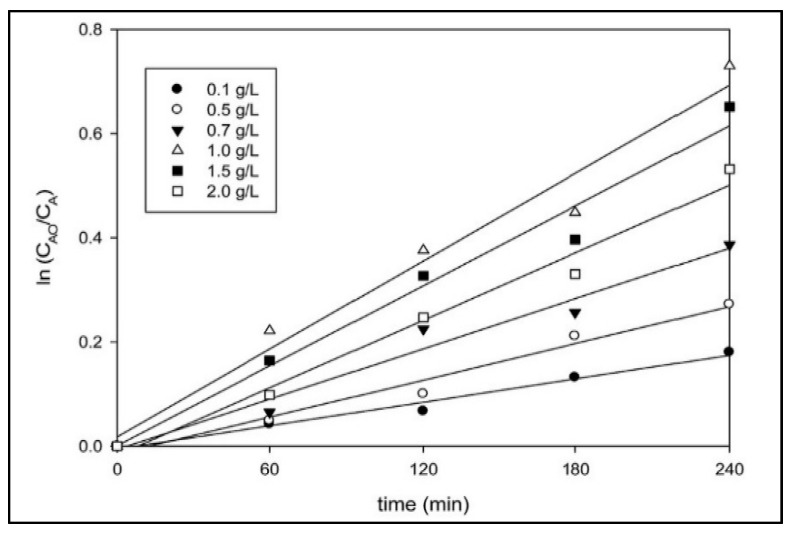
Reaction kinetic plots for photocatalytic degradation of POME. Reproduced from ref. [118] with permission from The Royal Society of Chemistry.

The equation can be written as follows:

(14) $$\mathrm{Zero}\;\mathrm{Order}\;(n=0):\;C_{0}-C=\mathrm{kt}$$

(15) $$\mathrm{First}\;\mathrm{Order}\;(n=1):\;\ln\left(\frac{C_{0}}{C}\right)=\mathrm{kt}$$

where C_0_ = initial reactant concentration, C = reactant concentration at time t, and t = time. By plotting the left term concerning time (t) of the two equations, the reaction order can be determined, while the value of k is obtained from the slope of the resulting curve. Ng and Cheng report the kinetics of the photocatalytic degradation of POME over UV-responsive TiO_2_ photocatalysts. It was found that the degradation kinetics of POME followed a 1^st^ order reaction with specific reaction rates (k) ranging from 0.70 × 10^−3^ to 2.90 × 10^−3^ min^−1^. Figure 21 shows the resulting modeling exercise. As a substitute for excellent linearity, it can be concluded that the decomposition of organic matter in POME does follow the first-order reaction.

## 9. Conclusions and Future Perspective

Palm oil industry waste (POME) has a high COD and BOD, which can cause environmental pollution and the death of life in water due to reduced oxygen levels. POME waste treatment using conventional technology such as an open pond system currently cannot completely decolorize POME waste. Besides, methane gas production released freely into the air can contribute up to 70% of total greenhouse gas emissions [30]. Various alternative POME treatment technologies have been carried out, although these technologies show a positive trend in dealing with POME waste. However, their high costs deter the deployment of these technologies for large-scale applications. In this regard, photocatalytic technologies may be an economically feasible alternative. The application of photocatalytic technology to convert palm oil industry (POME) waste has shown good potential on a laboratory scale [49,117,120,121,125,126,127]. Photocatalytic technology using either UV light or the sun is increasingly becoming a hot topic in research because it shows high efficiency in the mineralization of organic compounds and disinfection of pathogenic microorganisms in wastewater. A review of several studies of photocatalytic technology in POME waste treatment is summarized in Table 4.

However, the application of photocatalytic technology for POME waste treatment is limited by several key technical issues that need to be further investigated. The first consideration is whether the photocatalytic process in POME waste requires pretreatment or can be directly applied. It has been previously discussed that POME waste output has a high temperature (80–100 °C), while an increase in temperature in a photocatalytic reaction (>80 °C) will cause a decrease in photocatalytic activity. In addition, POME waste contains suspended solids, so preliminary treatment is needed to remove the solid suspension. This measure, of course, will require additional costs if applied on a large scale.

Several major technical obstacles ranging from catalyst development to process optimization must be overcome to promote photocatalytic technology feasibility in POME waste treatment soon. These include (i) developing photocatalysts for high photocatalytic efficiency that can utilize visible light or even a wider solar spectrum; (ii) the development of scalable photocatalyst synthesis methods in order to obtain the correct structure and size of the photocatalyst to increase photocatalytic efficiency; (iii) optimization in the parameters of photocatalytic operations needs to be investigated more fully based on the characteristics of POME waste. Currently, various efforts are being made to improve photocatalysts to work effectively, such as modification of the catalyst by doping to change the structure of the catalyst and energy bandgap. With photocatalysts powered by visible light or solar energy, we believe that photocatalysts can bridge the gap between lab-scale and large-scale production in POME waste’s photocatalytic treatment.

Overall, this review provides readers an overall idea about photocatalytic technology to reduce POME waste’s organic pollutants. With this systematic review text, the reader’s needs will be fulfilled properly, especially those new in photocatalytic technology for POME processing.

## Figures and Tables

**Table 1:** Characteristics of POME.

Parameter	Thani et al. [5]		Setiadi et al. [27]	
	Mean	Range	Mean	Range
pH *	4.2	3.4–5.2	4.1	3.3–4.6
Oil and Grease *	6000	150–18,000	-	-
BOD *	25,000	10,000–44,000	21,280	8200–35,400
COD *	50,000	16,000–100,000	34,720	15,103–65,100
Total Solids (TS) *	40,500	11,500–79,000	46,185	16,580–94,106
Suspended Solids (SS) *	18,000	5000–54,000	21,170	1330–50,700
Total Volatile Solid (TVS) *	34,000	90,00–72,000	-	-
Ammoniacal Nitrogen (AN) *	35	4–80	13	2.5–50
Total Nitrogen *	750	80–1400	41	12–126
Temperature (°C)	90	80–100	-	-

* All parameter units are in mg/L except pH and temperature.

**Table 2:** Effluent standards for the POME wastewater in Indonesia [8,28,29].

Year	1991		1995		2014	
Parameter	Highest Level (mg/L)	The Highest Pollution Load (kg/ton)	Highest Level (mg/L)	The Highest Pollution Load (kg/ton)	Highest Level (mg/L)	The Highest Pollution Load (kg/ton)
BOD_5_	250	1.5	250	1.5	100	0.25
COD	500	3.0	500	3.0	350	0.88
TSS	300	1.8	300	1.8	250	0.63
Oil and fat	30	0.18	30	0.18	25	0.063
Total Nitrogen (as N)	20	0.12	20	0.12	50	0.125
pH	6–9	-	6.0–9.0		6.0–9.0	
Highest waste discharge	-		6 m^3^ tons of raw material		2.5 m^3^ per ton of CPO	

**Table 3:** Summary of alternative POME treatment methods including COD removal efficiency, advantages, and disadvantages.

Treatment Methods	Type of Technology	Details	COD Removal (%)	Advantages	Disadvantages	Ref.
Biological treatment	Anaerobic	Upflow Anaerobic Sludge Blanket (UASB)	96	Produce methane gas Low energy demand and area requirement	Long startup phase	[73]
		Upflow Anaerobic Sludge Blanket-Hollow Centered Packed Bed (UASB-HCPB)	97.5	High methane production Useful for treatment of high suspended solid wastewater	Long startup phase Foaming at a high organic loading rate (OLR)	[12]
		Upflow Anaerobic Sludge Fixed Film Reactor (UASFF)	97	Produce methane gas Higher organic loading rate (OLR) achievable compared to operating UASB More stable operation.	Efficient in dilute POME	[54]
	Aerobic	Sequencing Batch Reactor (SBR)	96	High-quality effluent Simple single tank configuration Low cost Minimal sludge bulking	High energy for aeration No production of methane	[53]
		Rotating Biological Contactors (RBC)	88	Relatively low maintenance requirements Lower energy demand	Cannot handle high organic loading rate (OLR) Little flexibility in operating conditions	[55]
	Fermentation	Sequential two-stage	93	Achieve higher hydrogen yield	Cannot handle the high light intensity Only one enzyme is responsible	[74]
Physical treatment	Membrane technology	UF and RO	98.8	High potential for removing pollutants Offer water recycling	The membrane will experience fouling Requires high maintenance costs	[59]
	Adsorption	Chitosan	Oil removal: 99	Cleaner than biologically treated industrial waste, achieved in shorter maintenance times	Require further treatment	[61,63]
		Activated carbon	70	Cleaner than the industrial biologically treated effluent Shorter treatment time Reduction of agricultural waste disposal	Cannot handle high concentration	[15]
	Fenton-oxidation	Sono-Fenton	80	The sonication method is easy to use It does not produce sludge and residual gas	Requires costs for the purchase and operation of the sonicator unit Use an expensive probe	[66]
Chemical treatment	Coagulation-flocculation	Seed gum	48.2	Environmentally friendly Low cost	The high cost of sludge treatment Only effective in Total suspended solids removal	[58]
Thermo-chemical treatment	Steam reforming	Catalytic steam reforming	99	Syngas was successfully generated High COD and BOD removal	Carbon deposition on the catalyst surface High energy consumption.	[17,18]
Integration treatment	Biological Membrane	EGSB-Membrane	93	High-quality effluents It can be used as boiler feedwater	Costly treatment methods	[60]

**Table 4:** Previous studies of photocatalytic technology for degradation of POME waste.

Photocatalyst	Synthesis Method	Light Source	Degradation Rate	Catalyst Loading	Ref.
TiO_2_ nanoparticles (Degussa P25)	n.a. (Commercial)	UV B lamp	COD removal: 89% (5 h) TOD removal: 57% (5 h) Color reduction: 60% (5 h)	0.1 g/L	[115]
Cu/TiO_2_ (Degussa P25)	Impregnation	UV lamp (1000 W)	COD removal: 27% (1 h); >40% (7 h)	0.83 g/L (20 wt % Cu/TiO_2_)	[116]
TiO_2_	Sol-gel	UV Fluorescent tube (20 W)	COD removal: 97% (42 min) BOD removal: 95% (42 min) Decolorization: 92% (42 min)	0.01 g/L	[117]
TiO_2_ commercial	n.a. (Commercial)	UV lamp (100 W)	COD removal: 52% (4 h) COD removal: 78% (20 h)	1.0 g/L	[118]
TiO_2_ commercial	n.a. (Commercial)	UV lamp (100 W)	COD removal: 55% (4 h) BOD removal: 44% (4 h)	1.04 g/L	[119]
TiO_2_	n.a. (Commercial)	UV lamp (100 W)	COD removal: 52% (4 h); 80% (22 h)	1.0 g/L	[114]
ZnO			COD removal: 49% (4 h); 74% (22 h)		
TiO_2_ anatase	n.a. (Commercial)	Solar light	COD removal: 88% (5 h)	0.1 g/L	[120]
Pt/TiO_2_	Impregnation	UV lamp (100 W)	COD removal: 90% (8 h)	1.0 g/L (0.5 wt % Pt/TiO_2_)	[44]
		Xenon lamp (100 W)	COD removal: 11% (8 h)		
Ag/TiO_2_	Impregnation	UV lamp (100 W)	COD removal: 85% (8 h)	1.0 g/L (0.5 wt % Ag/TiO_2_)	[121]
		Xenon lamp (100 W)	COD removal: 60% (8 h)	1.0 g/L (0.5 wt % Ag/TiO_2_)	
Ag/TiO_2_	Impregnation	Visible lamp (250 W)	COD removal: 27% (8 h)	1.5 g/L (0.5 wt % Ag/TiO_2_)	[45]
CaFe_2_O_4_	Auto-combustion and coprecipitation	Xenon lamp (500 W)	COD removal: 56% (8 h)	1.0 g/L	[122]
CaFe_2_O_4_	Coprecipitation	Xenon lamp (500 W)	COD removal: 69% (8 h)	0.75 g/L	[123]
WO_3_ commercial	n.a. (Commercial)	UV lamp (100 W)	COD removal: 51% (4 h); 85% (16 h) Decolorization: 96% (4 h); 98% (16 h)	0.5 g/L	[106]
ZnO commercial	n.a. (Commercial)	Mercury lamp (100 W)	COD removal: 50% (4 h); 75% (22 h)	1.0 g/L	[124]
ZnO-PEG	Precipitation	UV lamp (15 W)	COD removal: 94% Decolorization: 84%	0.5 g/L	[125]
ZnO	Facile and surfactant-free reflux	Pen-ray UV-C (light intensity 5400 µW/cm^2^)	COD removal: 96% (2 h)	1.0 g/L	[126]
ZnO commercial	n.a. (Commercial)		COD removal: 69% (2 h)		
Nb_2_O_5_/ZnO	Surfactant-free chemical solution	UV lamp	COD removal: 92% (4 h) Decolorization: 100% (30 min)	3 wt % Nb_2_O_5_/ZnO	[127]

## Data Availability

No new data were created or analyzed in this study. Data sharing is not applicable to this article.
